# Drug Reaction With Eosinophilia and Systemic Symptoms (DRESS) Syndrome: A Case Report From South India

**DOI:** 10.7759/cureus.65128

**Published:** 2024-07-22

**Authors:** Srushti Sahukar, Vijay Chandrappa Byranahalli

**Affiliations:** 1 Internal Medicine, Nanjappa Hospital, Davangere, IND; 2 Anesthesiology and Critical Care, Nanjappa Hospital, Davangere, IND

**Keywords:** glutathione, elevated liver functions, drug hypersensitivity reactions, antiepileptic drug, drug reaction with eosinophilia and systemic symptoms (dress) syndrome

## Abstract

Drug Reaction with Eosinophilia and Systemic Symptoms (DRESS) syndrome usually presents two to six weeks after treatment with a drug implicated in this disorder. However, in some cases, it can present more than eight weeks after the initiation of an implicated medication. This is a type 4 drug hypersensitivity reaction in which any internal organ may be involved. While the liver is commonly involved, cardiac involvement is not unheard of. Comorbidities and multiorgan involvement may obscure the diagnosis, and Registry of Severe Cutaneous Adverse Reactions (RegiSCAR) criteria are a useful diagnostic aid. It is best treated by withdrawing the offending agent and administering systemic steroids. Oxidative stress is high in DRESS syndrome. Hepatoprotection is a priority in all patients and yields a better prognosis.

## Introduction

Drug Reaction with Eosinophilia and Systemic Symptoms (DRESS) syndrome is a potentially life-threatening idiosyncratic syndromal hypersensitivity reaction wherein the only definitive treatment is cessation of the offending agent [1-3]. We describe a case of DRESS syndrome that presented with multisystem findings and diagnostic ambiguity in a patient with several comorbidities, highlighting the critical role of timely diagnosis and management in improving patient outcomes. Mortality with DRESS syndrome can go as high as 10% [1,3]. Prompt treatment and cessation of the offending drug are of utmost importance. Our comprehensive treatment of the patient, including steroids, cardiac monitoring, and hepatoprotective agents, led to a marked clinical improvement and eventual recovery.

## Case presentation

A 50-year-old male patient arrived at our facility with fever, hypotension, tachypnea, shortness of breath, and a generalized maculopapular rash. He was transferred to our facility from a different setting, where he was diagnosed with acute coronary syndrome non-ST-elevation myocardial infarct with shock and treated as such with dual antiplatelet therapy. He had several comorbidities, including peripheral vascular disease, ischemic heart disease, right occipital ischemic stroke, scar epilepsy, and cerebral venous thrombosis. The temperature on arrival was 101°F. A rash was present on his back, trunk, extremities, and face. His arterial blood gas on arrival revealed elevated lactate and metabolic acidosis. Liver function tests revealed a cholestatic picture with an elevation of all four commonly tested liver enzymes (Table 1). The infectious disease workup included dengue and malarial antigen tests, which were negative (Table 2). A point-of-care ultrasound revealed an enlarged gallbladder. Very high troponin and procalcitonin levels raised concerns about acute coronary syndrome and septic shock (Table 1). Urine output was adequate.

**Table 1:** Laboratory findings ▲: elevated, ▼: reduced, L: liter, dL: deciliter, g: gram, mg: milligram, μg: microgram, ng: nanogram, pg: picogram, U: units, IU: international units

Laboratory findings	Day 0	Day 4	Reference range
Total bilirubin	1.91 ▲	1.31 ▲	0.2-0.7 mg/dL
Direct bilirubin	1.14 ▲	0.77 ▲	0-0.2 mg/dL
Indirect bilirubin	0.77	0.54	0.2-0.8 mg/dL
Gamma-glutamyl transferase	585 ▲	612 ▲	0-54 U/L
Aspartate aminotransferase	97.6 ▲	62▲	0-35 IU/L
Alanine aminotransferase	182 ▲	127 ▲	0-45 IU/L
Alkaline phosphatase	738 ▲	720 ▲	53-128 IU/L
Serum creatinine	1.48 ▲	1.3 ▲	0.6-1.2 mg/dL
Hemoglobin	12.3 ▼	10.90 ▼	14-18 mg/dL
Mean corpuscular hemoglobin	26.1 ▼	27.10	27-31 pg
Mean corpuscular hemoglobin concentration	30.8 ▼	32	31.5-34.5 g/dL
Platelet count	268	490 ▲	150-450 thousand/cubic millimeter
Total leucocyte count	21.02▲	3.67 ▼	4-11 thousand/cubic millimeter
Absolute neutrophil count	15.52▲	2.33	2-7 thousand/cubic millimeter
Absolute eosinophil count	2.74 ▲	0.11	0.04-0.4 thousand/cubic millimeter
Prothrombin time	14.9 ▲	14	9-14 seconds
Procalcitonin	>95.370 ▲		ng/mL (risk of severe sepsis or septic shock <0.5: low, 0.5-2: moderate, >2: high)
C-reactive protein	160.82 ▲		0-6 mg/dL
Troponin I	4591.8 ▲		0-40 ng/L
Random serum cortisol	3.32 ▼		5-24 μg/dL

**Table 2:** Dengue and malarial tests NS1: nonstructural protein 1, IgG and IgM: immunoglobulins G and M

Dengue and malarial tests (performed on day 0)	
Dengue	
NS1	Negative
IgG	Negative
IgM	Negative
Malaria	
Malarial antigen test	Negative

Treatment

Concerns of septic shock from high procalcitonin levels preceded the administration of broad-spectrum, empirical systemic antibiotic therapy with meropenem and linezolid to target both gram-positive and gram-negative organisms. Negative blood and urine cultures led us to de-escalate antibiotic therapy to cefoperazone-sulbactam. Suspicion of tropical fever led us to start the patient on doxycycline. Low-molecular-weight heparin was added for deep venous thrombosis prophylaxis. Ringer’s lactate was the fluid of choice for IV infusion. IV hydrocortisone 50 mg QID was started for relative adrenocortical insufficiency.

Suspicion for connective tissue diseases was ruled out with an antinuclear antibodies (ANA) profile and cytoplasmic and perinuclear antineutrophilic cytoplasmic antibodies (cANCA and pANCA) tests (Tables 3-4). The high elevation of troponins pointed to cardiac involvement. Dermatology opinion yielded DRESS syndrome as one of the differentials. Eight out of the 10 criteria mentioned in the Registry of Severe Cutaneous Adverse Reactions (RegiSCAR) specifications, along with three of the four starred criteria, were fulfilled (Table 5). This led to a collective decision by the team to stop the lamotrigine within 24 hours of arrival at our facility. He has switched to oral levetiracetam 500 mg BD and oral clobazam 5 mg OD for seizure prevention. Simultaneously, the patient was de-escalated from antibiotics.

**Table 3:** ANA profile ANA: antinuclear antibodies, MI-2: myositis-specific autoantibody, RNP: ribonucleoprotein, SS-A: anti–Sjögren's syndrome-related antigen A autoantibodies, SS-B: anti–Sjögren's syndrome-related antigen B autoantibodies, Ro-52: associated with systemic lupus erythematosus and Sjögren's syndrome, anti Scl-70: anti-topoisomerase I in scleroderma, dsDNA: double-stranded DNA, anti-Jo-1: anti-histidyl-tRNA synthetase, AMA-M2: anti-mitochondrial M2 antibody, DFS-70: anti-dense fine speckled 70, Ku: rare autoantibody seen in scleroderma-polymyositis overlap syndrome, Sm: anti-Smith antibody

ANA profile (performed on day 0)	
MI-2	Negative
RNP	Negative
SS-A	Negative
SS-B	Negative
Ro-52	Negative
Scl-70	Negative
dsDNA	Negative
Nucleosome	Negative
Histone	Negative
Centromere B	Negative
Jo-1	Negative
AMA-M2	Negative
DFS-70	Negative
Ku	Negative
Sm	Negative

**Table 4:** ANCA tests ANCA: antineutrophil cytoplasmic antibodies, cANCA: cytoplasmic ANCA, PR3: proteinase 3, pANCA: perinuclear ANCA, MPO: myeloperoxidase

ANCA ELISA IgG (performed on day 0)		
cANCA (PR3)	Negative	Positive = 20 RU/mL, negative <20 RU/mL
pANCA (MPO)	Negative	

**Table 5:** RegiSCAR criteria for diagnosis of DRESS RegiSCAR: Registry of Severe Cutaneous Adverse Reactions, DRESS: Drug Reaction with Eosinophilia and Systemic Symptoms Reference: Table derived from a paper by Choudhary et al. (2013) in The Journal of Clinical and Aesthetic Dermatology [1], with permission from authors to reproduce the table.

RegiSCAR criteria for diagnosis of DRESS
1. Fever >100.4°C*
2. Involvement of at least 1 organ system*
3. Enlarged lymph nodes in at least 2 sites*
4. Hematologic abnormalities*
5. Hospitalization
6. Acute rash
7. Suspicion of rash being drug-related
8. Lymphocytes above or below normal limits
9. Eosinophils above the normal limits
10. Platelets below the normal limits
Three out of the first four (asterisked) criteria are required for making the diagnosis.

The patient was treated with IV dexamethasone 8 mg TID as the mainstay for DRESS syndrome. For hepatoprotective effects, oral glutathione 500 mg BD, IV N-acetylcysteine (NAC) 1 g BD, and oral ursodeoxycholic acid 300 mg BD were administered. Clinical improvement was seen within three days after the discontinuation of the drug. Absolute eosinophil and neutrophil counts were normalized by day 4 of admission. The patient was discharged five days after admission on a prednisolone 50 mg dispersible tablet to be taken every morning, which was tapered after 10 days.

## Discussion

DRESS syndrome is a type 4 hypersensitivity reaction with a wide range of presentations that can make for a long list of differentials, which adds to the diagnostic challenge of an already rare syndrome [1,3]. A skin rash is very typical when DRESS syndrome is lamotrigine-induced and can be severe enough to cause toxic epidermal necrolysis [1,4]. The decision to put our patient on lamotrigine for focal seizures was with the intention of avoiding the slew of side effects with carbamazepine. Our patient has displayed a cholestatic liver injury, which happens to be a rarer presentation in DRESS syndrome [3]. Cardiac involvement was a prominent feature in our patient with an extreme elevation of cardiac troponins, for which cardiac monitoring was done very closely. Cardiac involvement in DRESS syndrome tends to be between 4% and 21% [5].

The management of DRESS syndrome mainly hinges on prompt cessation of the causative agent, supportive care, and steroid therapy [1-4]. Early cessation of the causative agent prevents irreparable liver damage, brings about disease resolution, and provides a mortality benefit [4]. Our patient showed symptomatic improvement within 24 hours of lamotrigine cessation. The quick diagnosis and the shrewdness of the team in assimilating the patient’s history and coming to the right conclusion were pivotal for this patient. It led to the early commencement of definitive treatment and an improved prognosis. Lamotrigine is one of the aromatic anticonvulsants, and the rash in our patient is typical of the anticonvulsant hypersensitivity syndrome (AHS) rash seen with this classification of drugs [1,4,6,7].

The effectiveness of steroid therapy is not established but is widely employed [1,3]. Oxidative stress in DRESS syndrome is a result of drug metabolism, immune cell activation, and the inflammatory response itself [8]. Lamotrigine is an aromatic anticonvulsant that is metabolized by the hepatic cytochrome P450 enzyme complex, a defect that can generate reactive oxygen species and cause cytotoxicity [9]. Glutathione is an excellent shield against oxidative stress, which is a major pathogenetic mechanism in DRESS syndrome [8,9]. Mortality in DRESS syndrome is commonly due to liver necrosis and/or failure, for which NAC, glutathione, and ursodeoxycholic acid have been employed for hepatoprotection [8-11].

The patient revealed that he was taking multiple medications every day, including certain homeopathic supplements, but was unable to elaborate on names and ingredients. Considering the patient’s preexisting stress of detoxification stemming from various medications he was taking to combat his comorbidities, we administered all three of the medications to minimize the likelihood of him developing hepatic necrosis. Recovery can take up to 3-10 months after drug cessation [12]. Our patient followed up with the neurologist in the outpatient department twice at one-month intervals after discharge and had no complaints or abnormalities on the physical exam.

Post-discharge, the patient refused liver function tests, despite our recommendation. A telephonic consultation 12 months after discharge revealed full recovery of functionality. The patient revealed that he is back at his job and has no current complaints.

Limitations

This paper talks about the treatment decisions taken for a single patient whose previous medical records were inaccessible to us and whose past medical history reliability was questionable due to the patient's low education level. The haphazard nature of the healthcare system in India, the absence of universal health insurance, and the high costs of testing to be paid by the patient out-of-pocket limited our ability to order an extensive workup.

## Conclusions

While it is important to consider DRESS syndrome in our differentials - the treatment of which is straightforward with respect to withdrawing the offending agent - it is also important to work up other differentials that might be complicating our arrival at the diagnosis. It is helpful to have a high index of suspicion, especially when the mainstay of treatment is so simple. Physicians must pay attention to the constellation of symptoms and improve their familiarity with this diagnostic entity. Hepatoprotective agents like ursodeoxycholic acid, glutathione, and NAC are excellent adjunctive medications for managing DRESS syndrome patients. Early diagnosis and supportive care are of utmost importance in improving prognosis.
